# Enhancing Oral Bioavailability and Brain Biodistribution of Perillyl Alcohol Using Nanostructured Lipid Carriers

**DOI:** 10.3390/ph16081055

**Published:** 2023-07-25

**Authors:** Samila Horst Peczek, Ana Paula Santos Tartari, Isabella Camargo Zittlau, Camila Diedrich, Christiane Schineider Machado, Rubiana Mara Mainardes

**Affiliations:** 1 Laboratory of Nanostructured Formulations, Universidade Estadual do Centro-Oeste, Alameda Élio Antonio Dalla Vecchia St., 838, Guarapuava 85040-167, PR, Brazil; samylahp@hotmail.com (S.H.P.); ap.tartari@hotmail.com (A.P.S.T.); isazittlau99@gmail.com (I.C.Z.); camiladiedrich@hotmail.com (C.D.); chrischineider@outlook.com (C.S.M.); 2 Department of Pharmacy, Universidade Estadual do Centro-Oeste, Alameda Élio Antonio Dalla Vecchia St., 838, Guarapuava 85040-167, PR, Brazil

**Keywords:** lipid nanoparticles, perillyl acid, pharmacokinetics, biodistribution

## Abstract

Perillyl alcohol (POH), a bioactive monoterpenoid derived from limonene, shows promise as an antitumor agent for brain tumor treatment. However, its limited oral bioavailability and inadequate brain distribution hinder its efficacy. To address these challenges, this study developed nanostructured lipid carriers (NLCs) loaded with POH to improve its brain biodistribution. The NLCs prepared using hot homogenization exhibited an average diameter of 287 nm and a spherical morphology with a polydispersity index of 0.143. High encapsulation efficiency of 99.68% was achieved. X-ray diffraction analyses confirmed the semicrystalline state of POH-loaded NLCs. In vitro release studies demonstrated a biphasic release profile. Stability studies in simulated gastric and intestinal fluids confirmed their ability to withstand pH variations and digestive enzymes. In vivo pharmacokinetic studies in rats revealed significantly enhanced oral bioavailability of POH when encapsulated in the NLCs. Biodistribution studies showed increased POH concentration in brain tissue with NLCs compared with free POH, which was distributed more in non-target tissues such as the liver, lungs, kidneys, and spleen. These findings underscore the potential of NLCs as effective delivery systems for enhancing oral bioavailability and brain biodistribution of POH, providing a potential therapeutic strategy for brain tumor treatment.

## 1. Introduction

Perillyl alcohol (POH; IUPAC name: [4-(prop-1-en-2-yl)cyclohex-1-en-1-yl]methanol) is a natural compound belonging to the monocyclic terpene group, which can be found in the essential oils of various plants and citrus fruits, including mint, cherries, lavender, lemongrass, celery seeds, ginger, and sage, among others [1]. Regarding its pharmacokinetics, POH exhibits a relatively short half-life, necessitating frequent dosing to maintain therapeutic plasma concentrations. After oral administration, the compound is rapidly absorbed, although its bioavailability may be limited due to extensive first-pass metabolism in the liver [2,3]. Notably, POH undergoes significant hepatic metabolism, leading to the formation of perilaldehyde, perillyl acid, and dihydroperillic acid. These metabolites are subsequently subjected to glucuronidation and primarily eliminated through the renal system, with a minor fraction excreted through the bile. These factors contribute to the pharmacokinetic limitations of POH [4,5,6].

Over the past few decades, there has been a substantial increase in the interest surrounding the medicinal properties of POH, driven by the discovery of its potent antitumor activity. Numerous in vitro and in vivo studies have demonstrated the chemopreventive effects of POH against various types of cancer, including lung [7], skin [8], liver [9], pancreas [10], colon [11], breast [12], leukemia cells [13], and glioblastomas [14]. Although the precise mechanisms by which POH exerts its antitumor effects are not fully elucidated, several significant mechanisms have been reported. These include the inhibition of Ras protein isoprenylation [15,16,17], modulation of AP-1 (activator protein-1) activity, induction of early G1 arrest and apoptosis, negative regulation of cyclin proteins, and targeting of various other cellular factors such as telomerase reverse transcriptase (hTERT), eukaryotic translation initiation factors eIF4E and eIF4G, sodium/potassium adenosine triphosphatase (Na/K-ATPase), Notch, nuclear factor kappa B (NF-κB), mammalian target of rapamycin (mTORC), M6P/IGF-II receptor genes, and transforming growth factor beta (TGFβ) [16,17,18].

Given its potential as an antitumor agent, POH has undergone a series of phase I and II clinical trials in patients with glioblastoma multiforme (GBM), a highly malignant grade IV astrocytic lineage glioma according to the WHO classification of CNS tumors [19]. While it demonstrated disease stabilization, oral administration of POH was associated with gastrointestinal toxicities. Increased side effects were reported with the use of high and frequent doses required to maintain the plasma concentration of POH and its metabolites at levels sufficient to induce the desired therapeutic effect [5,20,21].

Within this context, nanotechnology emerges as a compelling alternative for drug delivery, offering the capability to manipulate properties such as toxicological profiles, controlled release mechanisms, and targeted delivery to specific sites. In the field of oncology, the rapid advancements in nanotechnology have propelled nano-oncology as a promising approach for cancer therapy. The escalating interest in the application of nanotechnology in oncology primarily stems from the remarkable ability of nanomedicines to modify pharmacokinetic parameters and improve drug distribution at tumor sites, while concurrently reducing concentrations in healthy tissues. Consequently, these advancements have the potential to minimize undesirable side effects and address the limitations associated with conventional cancer treatments [22,23]. Our research group has successfully developed a nanoemulsion containing POH, which exhibited enhanced bioavailability and brain targeting when administered intranasally in rats [24]. However, we are now eager to explore an alternative nanoparticle system for the same purpose, with a specific focus on oral administration. This novel approach aims to overcome the limitations of intranasal delivery and provide a more convenient and patient-friendly route for the effective delivery of POH.

Among the various nanoparticulate drug delivery systems, lipid-based systems have emerged as a prominent field of research, with nanostructured lipid carriers (NLCs) receiving significant attention in recent decades. The supramolecular structure of NLCs is characterized by the arrangement of lipids in a nanostructured pattern. NLCs are comprised of a blend of solid and liquid lipids, exhibiting a core–shell structure. The composition of NLCs allows the incorporation of various lipids with diverse physicochemical properties, such as triglycerides, phospholipids, and fatty acids. This versatility enables the encapsulation of a wide range of hydrophobic and hydrophilic drugs, making NLCs suitable for delivering diverse therapeutic agents [25,26]. They contribute to the stability of the carriers, protect the encapsulated drugs from degradation, and facilitate controlled-release kinetics. Additionally, the supramolecular organization of NLCs influences their interactions with biological membranes and cellular uptake mechanisms, affecting their overall performance as drug delivery systems. Furthermore, NLCs exhibit scalability, enabling large-scale production to meet the demands of the pharmaceutical industry. Moreover, they possess sterilization potential, ensuring the maintenance of product quality and safety. These collective attributes make NLCs a versatile and promising platform for advanced drug delivery systems [27,28].

In this study, it was hypothesized that NLCs could enhance the oral bioavailability and brain biodistribution of POH, aiming to develop an effective oral formulation with reduced adverse effects for the treatment of glioblastoma. NLCs containing POH were synthesized and characterized, and their in vitro release properties were evaluated. Furthermore, the pharmacokinetic parameters and biodistribution were assessed following single oral administration in rats, providing valuable insights into the absorption, distribution, metabolism, and excretion of POH when encapsulated within NLCs. These comprehensive evaluations contribute to the understanding of the potential benefits of NLCs as a drug delivery system for improving the therapeutic outcomes of POH in glioblastoma treatment.

## 2. Results and Discussion

### 2.1. Preparation and Characterization of NLCs Containing POH

**Figure 1:**
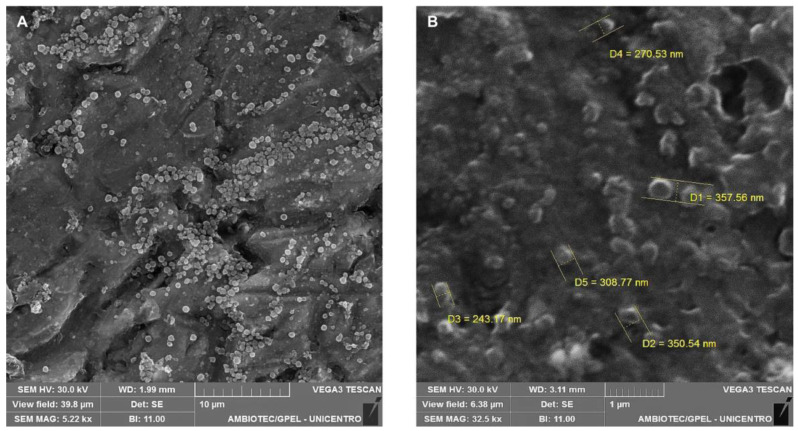
SEM images of POH-loaded NLCs at different magnification (**A**) 5.22 Kx and (**B**) 32.5 Kx.

The NLCs-POH were successfully obtained using the hot homogenization method. The methodology proved to be effective for NLCs’ production, as they exhibited appropriate size for oral delivery, low polydispersity, and excellent incorporation of POH (see Table 1), which served both as a structural component, being the liquid lipid of NLCs, and as the drug. SEM images (Figure 1A,B) revealed a relatively spherical morphology of the POH-loaded NLCs. Also, some aggregation was observed, however that may be attributed to drying processes involved in SEM sample preparation, which may not accurately represent the state of the particles in their dispersed form.

The mean diameter of the obtained NLCs was below 300 nm. Considering that tumor tissue vasculature has pores ranging from 200 to 1200 nm [29], the size of the NLCs is ideal for entering the fenestrations of tumor tissue neovasculature, while being unable to enter the narrow fenestrations of normal endothelium (10 to 50 nm), contributing to the enhanced permeability and retention (EPR) effect, leading to nanoparticle accumulation at the tumor site through passive targeting [30,31]. Furthermore, particles with a size ≤ 300 nm are more suitable for oral drug administration, as they are preferentially internalized by enterocytes and M cells and demonstrate increased intestinal transport compared with larger particles [32,33]. The choice of lipids with different physicochemical properties, such as chain length, degree of saturation, and melting point, can affect the lipid matrix arrangement and ultimately influence the size of the NLCs. Lipids with a higher melting point result in larger particle sizes due to the increased fusion viscosity in the system, consequently reducing the efficiency of the homogenization step for size reduction [34,35]. Studies have shown that NLCs prepared with Gelucire^®^ 43/01 exhibit smaller particle size compared with glyceryl tripalmitate [36], glyceryl behenate, and glyceryl monostearate [37], due to its relatively lower melting point. Moreover, the chemical composition of Gelucire^®^ 43/01, which contains mono- and diglycerides along with polyethylene glycol esters of fatty acids, provides certain emulsifying properties, facilitating emulsification and assisting in the formation of NLCs with smaller particle size [35,36,37].

The polydispersity index (PDI) serves as a measure of the uniformity in the size distribution of particles, reflecting the homogeneity or heterogeneity of the sample. It is a numerical value ranging from 0.0 (indicating a perfectly uniform sample) to 1.0 (representing a highly polydisperse sample with various particle sizes). Ideally, PDI values ≤ 0.2 indicate a more homogeneous size distribution, highlighting a desirable characteristic for nanoparticle formulations [38]. In the context of this study, the obtained NLCs exhibited a PDI value < 0.15 (see Table 1), falling within the desired range and indicating a relatively uniform particle size distribution. The achievement of such low PDI values can be attributed to the careful selection and combination of surfactants, which play a crucial role in stabilizing the NLCs and controlling their size and PDI. In this investigation, polysorbate 80 and soybean lecithin were employed as surfactant combination. It has been demonstrated in previous studies that utilizing a mixture of surfactants can lead to a reduction in PDI compared with using a single surfactant, thereby improving the overall stability of the NLC system [35,39]. Furthermore, the inclusion of non-ionic surfactants, such as polysorbate 80, has been shown to contribute to lower PDI values compared with anionic or cationic surfactants. This can be attributed to the ability of polysorbate 80 to decrease particle size and minimize aggregation tendencies, ultimately leading to a more uniform particle size distribution [40,41].

The obtained NLCs showed a zeta potential of −32.5 mV, indicating excellent predictability of formulation stability. This highly negative value suggests strong repulsive forces among the particles, effectively preventing internal phase aggregation [42]. The anionic nature of the solid lipid Gelucire^®^ 43/01 is responsible for conferring the negative charge, which contributes to the zeta potential observed. Encouragingly, similar zeta potential values (<−30 mV) have been reported in other investigations utilizing Gelucire^®^ 43/01 as the solid lipid for NLCs’ development [37,43]. The zeta potential not only ensures the stability and dispersion of NLCs, but also has implications for their interaction with biological systems. The electrostatic repulsion between NLC particles with such a significant negative charge can inhibit particle aggregation, enhance their colloidal stability, and promote their prolonged circulation time in biological fluids [44].

The entrapment efficiency (EE) of POH in NLCs was remarkably high, reaching almost 100%. The exceptional EE observed in this study can be attributed to the distinctive dual functionality of POH, as both a pharmacological agent and a structural element within the NLCs. As an oil, POH obviates the need for an additional liquid lipid component to form the supramolecular structure of the NLCs, ensuring its efficient encapsulation.

**Figure 2:**
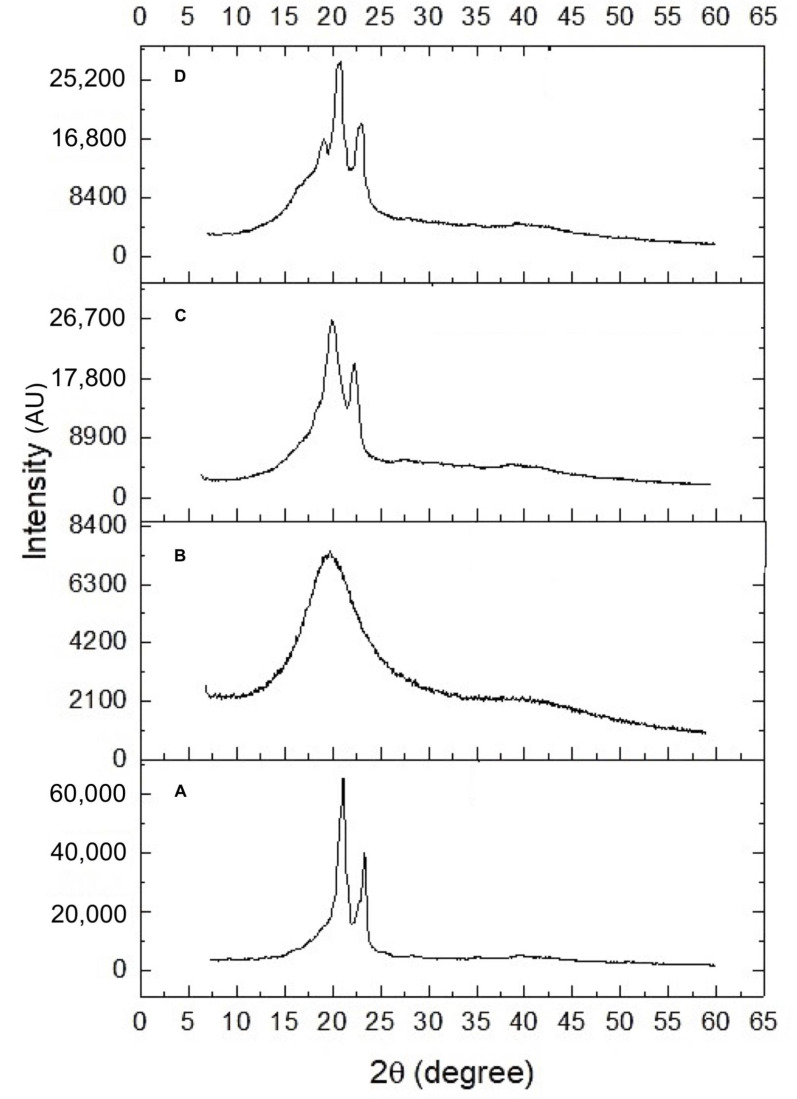
Diffractograms of: (**A**) Gelucire^®^ 43/01, (**B**) soybean lecithin, (**C**) blank NLCs, and (**D**) POH-loaded NLCs.

The diffractograms of the samples, including soybean lecithin, Gelucire^®^ 43/01, NLCs -POH, and blank nanoparticles (without POH), are presented in Figure 1. The XRD pattern of solid lipid Gelucire^®^ 43/01 (Figure 2A) exhibited characteristic peaks at 2θ: 20.9 and 23.1°, indicating its semicrystalline nature [45]. In contrast, the XRD pattern of soybean lecithin (Figure 2B) displayed a broad band at 20.3°, suggesting a completely amorphous profile with the absence of well-defined peaks [46]. The diffractograms of the blank NLCs (Figure 2C) and POH-loaded NLCs (Figure 2D) exhibited similar patterns, with a partial overlap of the characteristic Gelucire^®^ peaks at 2θ: 20.9 and 23.1°, although less distinct, and a broad base attributed to the lecithin’s broad band. This characterization indicates a semicrystalline profile of NLCs, which is anticipated to contribute to faster dissolution rates, improved absorption, and enhanced bioavailability [47].

### 2.2. In Vitro Release Profile

**Figure 3:**
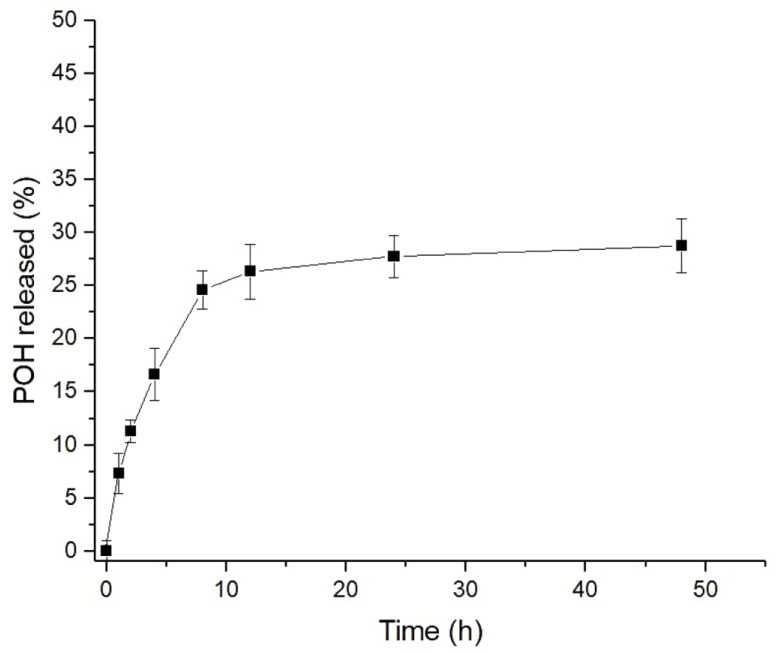
In vitro release of POH from NLCs in PBS pH 7.4, 37 °C (*n* = 3).

Figure 3 shows the in vitro release profile of POH from NLCs over a 48 h assay period. A prolonged release characterized by a biphasic profile was observed. An initial rapid release, known as the burst effect, was observed at 8 h, during which approximately 25% of POH was released from the NLCs. After this time, the release continued at a constant and slower rate, resulting in a cumulative release of 29% at the end of 48 h. The release profile obtained for POH was very similar to the one reported in the study by Zielińska et al. [48], using the same methodology, where the release of different monoterpenes, including limonene, a precursor of POH, was evaluated. Mathematical models were applied to the release data (Table 2), and the results showed that the best-fitted model (r = 0.922) was the Weibull model. This model suggests a drug release mechanism involving dissolution, diffusion, and mixed dissolution–diffusion rate-limited processes. The application of the semi-empirical Korsmeyer–Peppas model resulted in a release exponent of *n* = 0.40, indicating that drug release occurred through diffusion within the lipid matrix [49]. Thus, the initial burst release effect is attributed to the fraction of drug adsorbed on the NLCs’ surfaces, while Fickian diffusion drives sustained release curves according to the Weibull and Korsmeyer–Peppas models. Due to the inherent limitations of conducting in vitro release experiments with the physically oily nature of POH, it is expected that the release under in vivo conditions will exhibit a faster rate. Obviously, the pharmacokinetic assays will provide a comprehensive understanding of POH’s pharmacokinetic behavior that cannot be fully captured by in vitro experiments alone.

**Figure 4:**
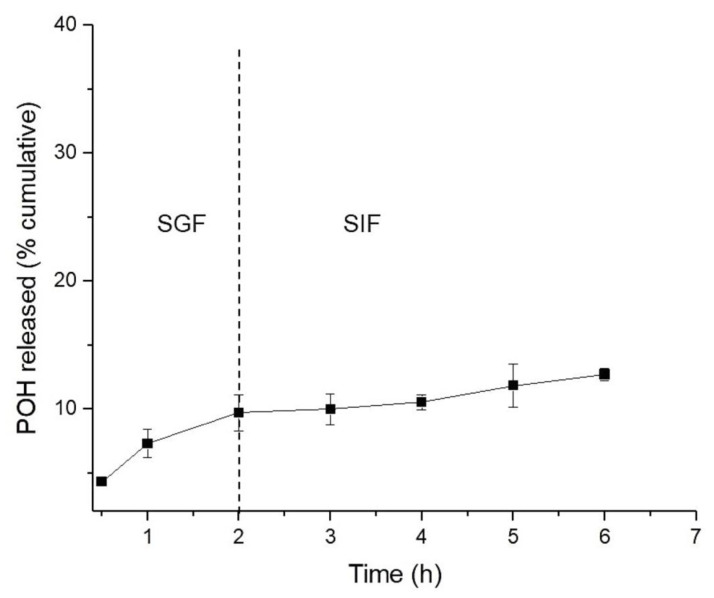
In vitro release of POH from NLCs in simulated gastric fluid (SGF) pH 1.2 and simulated intestinal fluid (SIF) pH 6.8, 37 °C (*n* = 3).

The release assay in simulated gastric fluid (SGF) and simulated intestinal fluids (SIF) was conducted to assess the suitability of the NLCs for oral administration. The study was conducted for 2 h in SGF pH 1.2, followed by a medium change to SIF pH 6.8 for 4 h. The release profile of POH from NLCs is presented in Figure 4. The NLCs showed low release of POH in both fluids, with a total of 12.4% released at the end of 6 h, suggesting excellent incorporation of the drug within the lipid matrix. These results indicate that the NLCs used for oral administration of POH demonstrated good stability, maintaining their integrity in the face of different pH variations and the presence of digestive enzymes. Therefore, the results suggest that the main content of POH incorporated in NLCs could be absorbed by intestinal cells and enter the bloodstream for sustained release in vivo. Moreover, this result is promising, considering that the oral administration of free-form POH caused significant gastrointestinal side effects in glioblastoma patients [20,21].

### 2.3. Pharmacokinetic Study

The pharmacokinetic study was conducted in rats to evaluate whether NLCs can alter the pharmacokinetic profile of POH. The absorption, bioavailability, and elimination profiles of orally administered POH-loaded NLCs were assessed in a single-dose study and compared with the pharmacokinetic profile obtained for free-form POH. Considering the properties of POH, such as low water solubility and low polarity, which pose challenges for its determination by ESI in the UPLC/MS-MS method, a decision was made to quantify the perillyl acid (PA), a metabolite of POH. Furthermore, due to the rapid metabolism of POH to PA, the levels of PA in plasma and brain were quantified instead [24,50].

**Figure 5:**
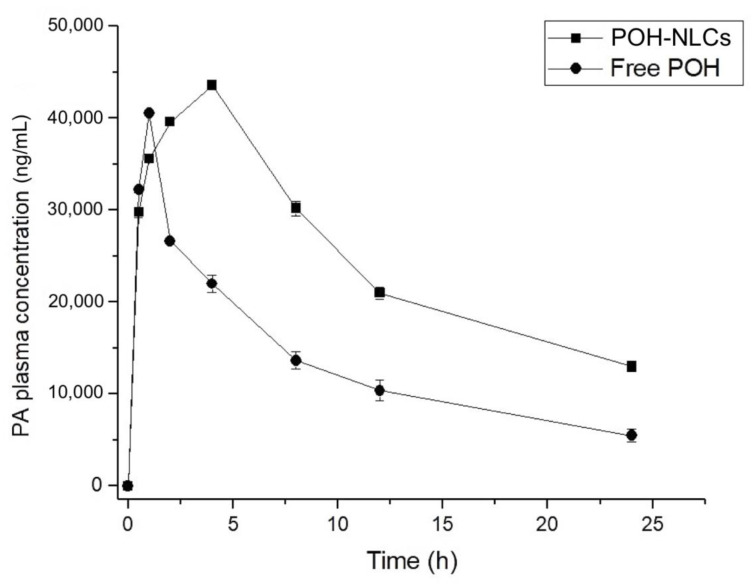
Plasma concentration–time curves of perillyl acid (PA) obtained after single oral administration of 500 mg/kg of POH in rats, using NLCs and in its free form.

**Figure 6:**
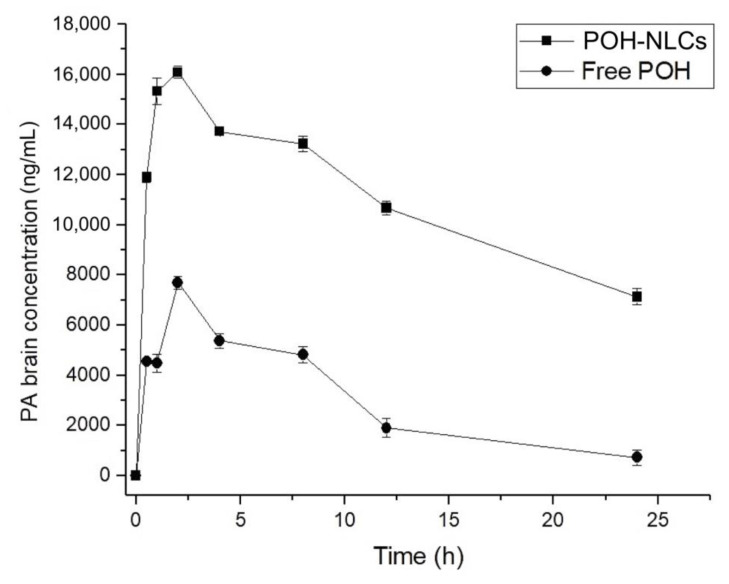
Brain concentration–time curves of perillyl acid (PA) obtained after single oral administration of 500 mg/kg of POH in rats, using NLCs and in its free form.

The mean plasma and brain concentration–time curves of PA following oral administration of 500 mg/kg of free-form POH and the equivalent of 500 mg/kg of POH in the NLCs are shown in Figure 5 and Figure 6, and the pharmacokinetic parameters are presented in Table 3 and Table 4.

Upon conducting an analysis of the plasma profile (Figure 5), it was evident that the oral administration of the free POH resulted in rapid absorption, with a maximum plasma concentration (C_max_) of 40,507.18 ng/mL observed at 1 h, followed by a decline to 5480.12 ng/mL after 24 h. In contrast, the NLCs exhibited a slower absorption rate, with a C_max_ of 43,552.17 ng/mL observed at 4 h and a sustained concentration of 12,999.93 ng/mL at the end of 24 h. This indicates a prolonged absorption period and sustained presence of the drug in the bloodstream when it is encapsulated in NLCs. Unlike the free drug, which is readily available for absorption, the drug encapsulated in the nanostructured delivery system undergoes lipid matrix dissociation, gradually releasing into the bloodstream. The oral bioavailability was enhanced two-fold (*p* < 0.05) with the utilization of NLCs in comparison to free-form POH treatment, as demonstrated by the increased AUC_0–24h_. Moreover, the half-life of the drug was prolonged from 8 to 11 h, indicating a reduced clearance rate and sustained presence in the systemic circulation.

The administration of NLCs significantly increased the brain concentration of PA compared to free-form POH treatment (Figure 6). This finding demonstrates that NLCs enhanced the absorption of POH, reaching a maximum concentration of PA (C_max_) of 16,085.07 ng/mL, which was approximately two times higher than the C_max_ of the free drug (7694.15 ng/mL) (*p* < 0.05). Furthermore, the prolonged duration of PA in the brain was evident, with a concentration of 7126.73 ng/mL at the end of 24 h, whereas the free-form POH had a PA final concentration of approximately 719.71 ng/mL. Analysis of the AUC_0–24h_ (Table 4) revealed a remarkable enhancement in the brain bioavailability of PA when the treatment was with NLCs, as its AUC_0–24h_ was increased 3.6-fold compared with the free drug. The efficacy of brain delivery can also be assessed by evaluating the AUCbrain/AUCplasma ratio of POH-loaded NLCs in comparison to free POH. The results revealed a ratio of 2, indicating that NLCs enhanced brain distribution by two-fold compared with the free drug. This ratio is correlated with a proportional improvement in bioavailability, also doubled, demonstrating the enhanced drug concentration in the brain. These results highlight the superior performance of NLCs in enhancing the brain uptake of PA and increasing its bioavailability in the brain.

In contrast, the distribution of free POH to organs such as the lungs, kidneys, liver, and spleen was more pronounced, indicating that NLCs displayed enhanced selectivity for brain delivery. This was evidenced by the AUC results (Table 4), where NLCs exhibited a higher accumulation of the drug in the brain while the free drug showed higher distribution to non-target tissues, such as liver, lungs, kidneys, and spleen. Furthermore, the plasma and brain half-life (T½) was prolonged in animals treated with NLCs, reinforcing the notion of sustained release mediated by these nanostructures and suggesting a prolonged therapeutic effect. These findings underscore the advantageous characteristics of NLCs in terms of improved tissue selectivity, extended drug release, and potential for enhanced therapeutic outcomes.

The precise mechanism underlying the transport of drugs across the blood–brain barrier (BBB) mediated by nanoparticles remains to be fully elucidated. Several potential mechanisms have been proposed to account for this phenomenon. These include the small size of nanoparticles, which provides a large surface area, as well as the lipophilic nature of NLCs. These factors enhance the interaction and prolonged contact time between the particles and the BBB, facilitating the transport of drugs to the brain by establishing a concentration gradient. The lipophilic properties of lipid nanoparticles enable their passage across the BBB through various transport pathways, such as paracellular, transcellular, transcytosis, and receptor-mediated endocytosis [51,52]. Further investigations are needed to gain a comprehensive understanding of the intricate mechanisms involved in lipid nanoparticle-mediated drug delivery to the brain.

Several studies have highlighted the role of polysorbate-80 in brain targeting, as it exerts specific effects on membrane fluidity, enhances interaction with the BBB, and inhibits efflux pumps like P-glycoprotein (P-gp) expressed in brain endothelial cells. These actions contribute to increased brain delivery of nanoparticles. Another significant mechanism involves the covalent association of polysorbate-80 with apolipoprotein E in the bloodstream. Apolipoprotein E is essential for the transportation of low-density lipoprotein (LDL) to the brain. By binding to the nanoparticle surface, polysorbate-80 enables the nanoparticles to mimic LDL particles, facilitating their interaction with LDL receptors. This interaction promotes the uptake of nanoparticles by cerebral capillary endothelial cells and enhances drug distribution within the brain compared with the free drug solution [53,54]. In this study, the presence of polysorbate-80 on the surface of NLCs indicates that the enhanced brain bioavailability may be attributed to its role in facilitating nanoparticle uptake via endocytosis, in addition to the previously discussed factors such as improved plasma bioavailability and prolonged release properties.

The findings of this study provide valuable insights into the pharmacokinetic behavior of the NLCs encapsulating the drug POH. The observed differences in the absorption, biodistribution, and elimination profiles between the POH-loaded NLCs and the free drug highlight the potential of nanotechnology in modulating drug pharmacokinetics for improved therapeutic outcomes. The sustained release, improved bioavailability, and enhanced brain bioavailability observed with NLCs offer promising prospects for improving the treatment of brain-related diseases. In the case of POH, a candidate for the treatment of glioblastoma multiforme, providing patients with an alternative that minimizes the frequency of administration and enhances action in the affected organ is of great importance. This increased selectivity of NLCs for brain delivery can help minimize potential adverse effects in non-target organs and improve the overall safety profile of the drug. Continued research and development in this field hold great potential for advancing therapeutic strategies and addressing the challenges associated with drug delivery to the brain.

## 3. Materials and Methods

### 3.1. Materials

Gelucire^®^ 43/01 (a mixture of mono-, di-, and triglyceride esters of fatty acids) and soy lecithin were kindly donated by Prati-Donaduzzi pharmaceutical company (Toledo, Brazil). Formic acid (98–100% purity), perillyl acid (PA, 95% purity), perillyl alcohol (POH, ≥95% purity), L-carvone (96% purity), pancreatin (99%), Pepsin (99%), and Tween 80—polysorbate 80 were obtained from Sigma-Aldrich^®^ (St. Louis, MO, USA). Potassium chloride (99%), sodium chloride (99%), and anhydrous monobasic sodium phosphate (99%) were acquired from Biotec^®^ (São José dos Pinhais, Brazil). HPLC-grade acetonitrile was purchased from Honeywell^®^ (NJ, USA). Dibasic sodium phosphate (99%) was obtained from Synth^®^ (São Paulo, Brazil). Purified water was obtained using a Milli-Q Plus system (Millipore Corporation, Bedford, MA, USA) with a conductivity of 18 MΩ.

### 3.2. Preparation of Nanostructured Lipid Carriers (NLCs) Containing POH

The NLCs containing POH were obtained using the hot homogenization method, according to Zhuang et al. (2010) [55], with modifications. It is noteworthy that POH, in addition to being the drug, also served a structural function as the liquid lipid component of the NLCs. In brief, the oily phase composed of POH (700 μL) and the solid lipid Gelucire^®^ 43/01 (1.3 g) was heated to 53 °C (10 °C above the melting temperature of the solid lipid) for 10 min. Separately, 10 mL of an aqueous solution containing polysorbate 80 (1%) and soy lecithin (1%) was heated to the same temperature and added to the melted oily phase, followed by agitation using an Ultra Turrax homogenizer (Dremel^®^, 300, São Paulo, Brazil) at 33,000 RPM for 2 min. The obtained pre-emulsion was subjected to sonication using an ultrasonic sonicator (Eco-Sonics^®,^ Indaiatuba, Brasil) for 2 cycles of 1 min each, at a power of 90 Watts, and subsequently cooled (2–8 °C) for 5 min to allow the formation of the NLCs. Afterwards, NLCs underwent ultracentrifugation at 25,240× *g* and 4 °C for 20 min (Hermle—Z36HKl centrifuge, Wehingen, Germany). Subsequently, the resulting precipitate was redispersed in ultrapure water, while the supernatant was retained for subsequent analysis.

### 3.3. Physicochemical Characterization

The mean size and polydispersity index (PDI) of the NLCs were determined using dynamic light scattering (BIC 90 plus, Brookhaven Inst. Corp., Holtsville, NY, USA). The samples were diluted in ultrapure water and subjected to analysis using a scattering angle of 90 degrees and a laser beam with a wavelength of 695 nm. The zeta potential was measured based on the electrophoretic mobility of the nanoparticles (Zetasizer ZS, Malvern, UK). The morphological features were evaluated using a scanning electron microscope (SEM) operated at an acceleration voltage of 30 kV (VEGA3, Tescan Orsay Holding, Brno, Czech Republic). X-ray diffraction (XRD) analysis was performed using an X-ray diffractometer (D2 Phaser, Bruker, Mannheim, Germany) equipped with Cu Kα radiation (λ = 1.5418 Å) at 30 kV voltage and 10 mA current. The samples were placed on a glass support and analyzed in the 2θ open-angle range of 5 to 60°.

### 3.4. Entrapment Efficiency Determination

The entrapment efficiency (EE) was determined indirectly by quantifying the amount of POH present in the supernatant obtained after the NLCs’ ultracentrifugation. This was achieved using a validated high-performance liquid chromatography (HPLC) method. A sample of the supernatant was taken and diluted in the mobile phase before being filtered through a 0.22 μm pore-size filter. The HPLC analysis was conducted using a Waters^®^ 600 Alliance system with a diode array detector (DAD) model 2696. A C18 column (5 μm, 4.6 mm × 250 mm—AtlantisTM) was utilized. The chromatographic conditions involved a mobile phase composition of acetonitrile:acidified water (0.5% formic acid) (60:40, *v*/*v*). The separation was performed using isocratic elution at a flow rate of 1.0 mL/min, with the detection wavelength set at 193 nm for POH. Subsequently, the EE% was calculated according to Equation (1):

EE (%) = (POHi − POHs)/POHi × 100 (1)

where initial POHi represents the initial quantity of the POH incorporated into the NCLs, and POHs refers to the portion of the POH that remains unincorporated in the supernatant of NLCs.

### 3.5. In Vitro Release Profile

The in vitro release of POH from NLCs was assessed using Franz diffusion cells (Hanson Corp., Chatsworth, CA, USA). The receptor medium, phosphate-buffered saline (PBS, 50 mM, pH 7.4), was maintained at a temperature of 37 ± 0.5 °C with continuous stirring at 400 rpm. A cellulose acetate membrane with a pore-size cutoff of 0.22 μm was interposed between the donor and receptor compartments. A 20 μL suspension of NLCs-POH, ensuring sink conditions, was applied onto the membrane. At predetermined time intervals (0.5, 1, 2, 4, 8, 12, 24, and 48 h), 1.0 mL aliquots were collected and subsequently analyzed using HPLC.

Furthermore, the in vitro release of POH was evaluated in simulated gastric fluid (SGF) and simulated intestinal fluid (SIF), utilizing the dialysis membrane technique. In this experiment, a 500 μL aliquot of the loaded POH was placed inside a dialysis bag (MWCO 14,000, INLAB^®^, São Paulo, Brazil) and dispersed in 2 mL of pH 7.4 PBS. The bag was immersed in reservoirs containing 60 mL of pH 1.2 SGF supplemented with pepsin for a period of 2 h. Subsequently, the medium was replaced with pH 6.8 simulated SIF containing pancreatin and incubated for an additional 4 h. The release media were maintained at 37 ± 0.5 °C with magnetic stirring at 500 rpm. At specific time points (0.5, 1, 2, 3, 4, 5, and 6 h), aliquots were collected, and the withdrawn volume was replenished with fresh SGF or SIF. The collected samples were filtered through 0.22 μm PVDF membranes and subjected to analysis using HPLC.

### 3.6. Pharmacokinetic Study

#### 3.6.1. UPLC-MS/MS Analysis

Ultra-performance liquid chromatography (UPLC) coupled with a triple quadrupole mass spectrometer (XEVO-TQD, Waters^®^, Milford, MA, USA) equipped with a electrospray ionization source (Waters^®^, Milford, MA, USA) was employed to determine and quantify the presence of perillyl acid (PA, a metabolite of POH) in rat plasma and organs following treatment. The chromatographic conditions included a reverse-phase C18 column (100 mm × 2.1 mm) with a mobile phase composed of acetonitrile (ACN) and acidified water (0.1% formic acid) in a ratio of 70:30 (*v*/*v*). The mobile phase was delivered isocratically at a flow rate of 0.3 mL/min, and a 2 μL injection volume was used. The total run time was 3 min, with retention times of 1.14 min for PA and 1.31 min for the internal standard (IS), carvone. The column oven was maintained at 40 °C, while the samples were kept at 10 °C in the autosampler. Electrospray ionization in positive mode (ESI+) was employed for mass spectrometric detection of the analytes. The MS conditions were as follows: capillary voltage at 4 kV, source temperature at 150 °C, desolvation temperature at 500 °C, and desolvation gas flow rate at 800 L/h. Quantification was performed using multiple reaction monitoring (MRM) mode, with *m*/*z* 167 → 92.97 for PA and *m*/*z* 151.01 → 122.98 for the IS. The optimized collision energies were 16 eV for PA and 8 eV for the IS. Instrument control, data acquisition, and processing were carried out using MassLynx™ 4.1 software (Milford, MA, USA).

#### 3.6.2. Treatment

The pharmacokinetic study was approved by the Animal Ethics Committee of the Universidade Estadual do Centro-Oeste, Brazil (registration number 025/2021). It used adult male Wistar rats weighing between 200 and 300 g. The animals were housed in cages with ad libitum access to water and food, following a 12 h light/dark cycle. To minimize the influence of food, a fasting period of 12 h was implemented prior to drug administration in all rats. However, unrestricted access to water was provided throughout the study. The animal subjects were allocated into two distinct groups: Group A, which received free POH, and Group B, which received a suspension of NLCs encapsulating POH. In both groups, POH was administered orally via gavage as a single dose equivalent to 500 mg/kg. After the designated treatment duration, the animals were anesthetized (using Ketamine at a dose of 75 mg/kg and Xylazine at a dose of 10 mg/kg, administered intraperitoneally) and euthanized by decapitation at predefined time points (0.5, 1, 2, 4, 8, 12, and 24 h). Each of the seven time intervals in Groups A and B involved the use of six animals. Subsequent to euthanasia, blood samples were collected, and the following organs were extracted from the animals: brain, lungs, kidneys, spleen, and liver.

#### 3.6.3. Sample Preparation

The collected blood samples were transferred to heparinized microtubes and centrifuged at 5000 rpm, 4 °C for 15 min to extract the plasma. Subsequently, liquid–liquid extraction with acetonitrile was performed. For this purpose, a 50 µL aliquot of plasma sample was added to 250 μL of acetonitrile containing the IS carvone (100 ng/mL). The tubes were then centrifuged at 15,000 rpm for 10 min at 4 °C. The supernatant was filtered through 0.22 µm syringe filters (Filtrilo, PVDF, São Paulo, Brazil) and transferred to an injection vial.

The organ homogenates were obtained by adding 5 mL of PBS (pH 7.4) to each 1 g of tissue, followed by homogenization at 33,000 rpm for 1 min for 2 cycles. A 250 μL aliquot of the homogenate was taken, and 750 μL of acetonitrile with the internal standard (100 ng/mL) was added. The samples were then centrifuged at 5000 rpm for 10 min at 4 °C and subsequently filtered through a 0.22 μm PVDF filter and analyzed using UPLC-MS/MS.

#### 3.6.4. Data Analysis

The pharmacokinetic parameters analyzed included peak plasma concentration (C_max_), time to reach peak plasma concentration (T_max_), area under the plasma concentration versus time curve (AUC_0–24h_), elimination half-life (T_½_), elimination rate constant (Kel), apparent volume of distribution (Vd), and clearance (Cl).

### 3.7. Statistical Analysis

Data of all the experiments were expressed as the mean value ± SD. One-way ANOVA with Tukey test was used to compare means at the statistical significance level of 95% level of confidence, *p* < 0.05 (Statistica v. 12 StatSoft Inc., Tulsa, OK, USA).

## 4. Conclusions

This study investigated the pharmacokinetic profile of a nanostructured drug delivery system consisting of NLCs encapsulating the drug POH. The findings demonstrate notable advantages of the formulation compared with the free drug form. Specifically, the NLCs exhibited prolonged absorption time and elevated drug concentration in the bloodstream, indicating enhanced systemic exposure. Moreover, the NLCs demonstrated enhanced selectivity for brain delivery, leading to significantly higher levels of the drug in the brain compared with the free drug. This represents a significant advantage over the free drug, which exhibits faster clearance and distribution to non-target tissues. This increased brain bioavailability of POH-loaded NLCs offers promising prospects for the treatment of glioblastoma multiforme, where efficient drug delivery to the brain is crucial. Looking ahead, several perspectives emerge from this study. Firstly, further investigations are warranted to elucidate the underlying mechanisms responsible for the enhanced brain delivery observed with the NLC formulation. Understanding the specific interactions between the NLCs and the blood–brain barrier can offer valuable insights into optimizing and tailoring the delivery system for improved efficacy. Additionally, exploring the therapeutic efficacy of the POH-loaded NLCs in preclinical and clinical studies, particularly in glioblastoma models, would provide essential evidence for their potential as a viable treatment strategy. Lastly, investigating the long-term stability, scalability, and manufacturing feasibility of the NLC formulation will be crucial for its future translation into clinical applications.

## Figures and Tables

**Table 1:** Characteristics of NLCs incorporating POH.

Characteristic	Result (Mean ± SD)
Mean particle size (nm)	288 ± 23
Polydispersity index	0.143 ± 0.040
Zeta potential (mV)	−32.5 ± 1.4
Entrapment efficiency (%)	99.6 ± 0.4

**Table 2:** Kinetic analysis of POH release from NLCs in 50 mM PBS solution, pH 7.4, 37 °C.

Kinetic Model	*r* (Correlation Coefficient)
Zero order	0.554
First order	0.449
Second order	0.337
Third order	0.247
Higuchi	0.277
Korsmeyer–Peppas	0.916
Weibull	0.922
Hickson–Crowell	0.486

**Table 3:** Pharmacokinetic plasma parameters of perillyl acid, following oral administration of a single dose of 500 mg/kg of free POH and POH-loaded NLCs.

Parameter	Free POH	POH-Loaded NLCs
C_max_ (ng.mL^−1^)	40,507.18	43,552.17
T_max_ (h)	1	4
AUC_0–24h_ (ng.h/mL)	322,833.78	598,139.18
T_½_ (h)	8.65	11.90
Kel (1/h)	0.080	0.058
Cl (L/h)	0.0015	0.0008
Vd (L)	0.0193	0.0143

C_max_: maximum concentration; T_max_: time to reach maximum concentration; AUC_0–24h_: area under the plasma concentration–time curve; T_1/2_: half-life; Kel: elimination rate constant; Vd: apparent volume of distribution; Cl: clearance.

**Table 4:** Pharmacokinetic tissues parameters of perillyl acid, following oral administration of a single dose of 500 mg/kg of free POH and POH-loaded NLCs.

Treatment	Tissue	C_max_ (ng.mL^−1^)	T_max_ (h)	AUC_0–24h_ (ng.h/mL)	T_½_ (h)	Kel (1/h)
	Brain	7694.15	2	72,104.51	6.40	0.108
	Lung	41,265.63	2	442,244.37	8.63	0.080
Free POH	Kidney	34,245.26	2	437,725.63	12.92	0.053
	Liver	40,490.68	4	678,190.26	29.11	0.023
	Spleen	13,022.72	4	225,678.26	8.68	0.079
	Brain	16,085.07	2	263,771.20	19.35	0.035
	Lung	19,067.11	1	192,494.69	12.15	0.057
POH-loaded NLCs	Kidney	23,873.44	1	228,540.18	10.10	0.068
	Liver	28,884.92	2	320,092.07	19.74	0.035
	Spleen	10,662.32	1	75,687.80	5.52	0.125

C_max_: maximum concentration; T_max_: time to reach maximum concentration; AUC_0–24h_: area under the tissue concentration–time curve; T_1/2_: half-life; Kel: elimination rate.

## Data Availability

The data presented in this study are available on request from the corresponding author.

## References

[1] Shojaei S., Kiumarsi A., Moghadam A.R., Alizadeh J., Marzban H., Ghavami S. (2014). Perillyl Alcohol (Monoterpene Alcohol), Limonene. Enzymes.

[2] Hernandez O., Cenis J.L., Ferragut J.A., Seco E.M. (2011). Antitumor Effects of Perillyl Alcohol in the Malignant Cells Derived from Glioblastoma Multiforme: An In Vitro and In Vivo Preclinical Study. Investig. New Drugs.

[3] de Lima D.C., Rodrigues S.V., Boaventura G.T., Cho H.-Y., Chen T.C., Chonthal A.H., Da Fonseca C.O. (2020). Simultaneous measurement of perillyl alcohol and its metabolite perillic acid in plasma and lung after inhalational administration in Wistar rats. Drug Test. Anal..

[4] Chen T.C., Da Fonseca C.O., Schönthal A.H. (2018). Intranasal Perillyl Alcohol for Glioma Therapy: Molecular Mechanisms and Clinical Development. Int. J. Mol. Sci..

[5] Chen T.C., Da Fonseca C.O., Schönthal A.H. (2015). Preclinical development and clinical use of perillyl alcohol for chemoprevention and cancer therapy. Am. J. Cancer Res..

[6] Hudes G.R., Szarka C.E., Adams A., Ranganathan S., McCauley R.A., Weiner L.M., Langer C.J., Litwin S., Yeslow G., Halberr T. (2000). Phase I pharmacokinetic trial of perillyl alcohol (NSC 641066) in patients with refractory solid malignancies. Clin. Cancer Res..

[7] Xu M., Floyd H.S., Greth S.M., Chang W.-C.L., Lohman K., Stoyanova R., Kucera G.L., Kute T.E., Willingham M.C., Miller M.S. (2004). Perillyl alcohol-mediated inhibition of lung cancer cell line proliferation: Potential mechanisms for its chemotherapeutic effects. Toxicol. Appl. Pharmacol..

[8] Barthelman M., Chen W., Gensler H.L., Huang C., Dong Z., Bowden G.T. (1998). Inhibitory effects of perillyl alcohol on UVB-induced murine skin cancer and AP-1 transactivation. Cancer Res..

[9] Mills J.J., Chari R.S., Boyer I.J., Gould M.N., Jirtle R.L. (1995). Induction of apoptosis in liver tumors by the monoterpene perillyl alcohol. Cancer Res..

[10] Stark M., Burke Y.D., McKinzie J.H., Ayoubi A., Crowell P.L. (1995). Chemotherapy of pancreatic cancer with the monoterpene perillyl alcohol. Cancer Lett..

[11] Reddy B.S., Wang C.X., Samaha H., Lubet R., Steele V.E., Kelloff G.J., Rao C.V. (1997). Chemoprevention of colon carcinogenesis by dietary perillyl alcohol. Cancer Res..

[12] Yuri T., Danbara N., Tsujita-Kyutoku M., Kiyozuka Y., Senzaki H., Shikata N., Kanzaki H., Tsubura A. (2004). Perillyl Alcohol Inhibits Human Breast Cancer Cell Growth in vitro and in vivo. Breast Cancer Res. Treat..

[13] Clark S.S., Zhong L., Filiault D., Perman S., Ren Z., Gould M., Yang X. (2003). Anti-leukemia effect of perillyl alcohol in Bcr/Abl-transformed cells indirectly inhibits signaling through Mek in a Ras- and Raf-independent fashion. Clin. Cancer Res..

[14] da Fonseca C.O., Simão M., Lins I.R., Caetano R.O., Futuro D., Quirico-Santos T. (2011). Efficacy of monoterpene perillyl alcohol upon the survival rate of patients with recurrent glioblastoma. J. Cancer Res. Clin. Oncol..

[15] Crowell P.L., Ren Z., Lin S., Vedejs E., Gould M.N. (1994). Structure-activity relationships among monoterpene inhibitors of protein isoprenylation and cell proliferation. Biochem. Pharmacol..

[16] da Fonseca C.O., Landeiro J.A., Clark S.S., Quirico-Santos T., Carvalho M.d.G.d.C., Gattass C.R. (2006). Recent advances in the molecular genetics of malignant gliomas disclose targets for antitumor agent perillyl alcohol. Surg. Neurol..

[17] da Fonseca C.O., Santos T.Q., Fernandes J., da Costa Carvalho MD G., Gatass C.R. (2003). Biologia molecular dos glioblastomas: Perspectivas terapêuticas do monoterpeno álcool perílico. J. Bras. Neurocir..

[18] Chen T.C., da Fonseca C.O., Levin D., Schönthal A.H. (2021). The Monoterpenoid Perillyl Alcohol: Anticancer Agent and Medium to Overcome Biological Barriers. Pharmaceutics.

[19] Louis D.N., Perry A., Reifenberger G., Von Deimling A., Figarella-Branger D., Cavenee W.K., Ohgaki H., Wiestler O.D., Kleihues P., Ellison D.W. (2016). The 2016 World Health Organization Classification of Tumors of the Central Nervous System: A summary. Acta Neuropathol..

[20] Bailey H.H., Wilding G., Tutsch K.D., Arzoomanian R.Z., Alberti D., Feierabend C., Simon K., Marnocha R., Holstein S.A., Stewart J. (2004). A phase I trial of perillyl alcohol administered four times daily for 14 days out of 28 days. Cancer Chemother. Pharmacol..

[21] Bailey H.H., Levy D., Harris L.S., Schink J.C., Foss F., Beatty P., Wadler S. (2002). A Phase II Trial of Daily Perillyl Alcohol in Patients with Advanced Ovarian Cancer: Eastern Cooperative Oncology Group Study E2E96. Gynecol. Oncol..

[22] Chaturvedi V.K., Singh A., Singh V.K., Singh M.P. (2018). Cancer Nanotechnology: A New Revolution for Cancer Diagnosis and Therapy. Curr. Drug Metab..

[23] Diedrich C., Zittlau I.C., Machado C.S., Fin M.T., Khalil N.M., Badea I., Mainardes R.M. (2022). Mucoadhesive nanoemulsion enhances brain bioavailability of luteolin after intranasal administration and induces apoptosis to SH-SY5Y neuroblastoma cells. Int. J. Pharm..

[24] Santos J.D.S., Diedrich C., Machado C.S., da Fonseca C.O., Khalil N.M., Mainardes R.M. (2021). Intranasal administration of perillyl alcohol–loaded nanoemulsion and pharmacokinetic study of its metabolite perillic acid in plasma and brain of rats using ultra-performance liquid chromatography/tandem mass spectrometry. Biomed. Chromatogr..

[25] Khosa A., Reddi S., Saha R.N. (2018). Nanostructured lipid carriers for site-specific drug delivery. Biomed. Pharmacother..

[26] Tapeinos C., Battaglini M., Ciofani G. (2017). Advances in the design of solid lipid nanoparticles and nanostructured lipid carriers for targeting brain diseases. J. Control. Release.

[27] Müller R.H., Mäder K., Gohla S. (2000). Solid lipid nanoparticles (SLN) for controlled drug delivery: A review of the state of the art. Eur. J. Pharm. Biopharm..

[28] Mehnert W., Mäder K. (2001). Solid lipid nanoparticles: Production, characterization and applications. Adv. Drug Deliv. Rev..

[29] Aslan B., Ozpolat B., Sood A.K., Lopez-Berestein G. (2013). Nanotechnology in cancer therapy. J. Drug Target.

[30] Maeda H., Greish K., Fang J. (2006). The EPR Effect and Polymeric Drugs: A Paradigm Shift for Cancer Chemotherapy in the 21st Century. Adv. Polym. Sci..

[31] Antonio E., Dos Reis Antunes Junior O., Marcano R., Diedrich C., da Silva Santos J., Machado C., Khalil N., Mainardes R. (2021). Chitosan modified poly (lactic acid) nanoparticles increased the ursolic acid oral bioavailability. Int. J. Biol. Macromol..

[32] He C., Yin L., Tang C., Yin C. (2012). Size-dependent absorption mechanism of polymeric nanoparticles for oral delivery of protein drugs. Biomaterials.

[33] Banerjee A., Qi J., Gogoi R., Wong J., Mitragotri S. (2016). Role of nanoparticle size, shape and surface chemistry in oral drug delivery. J. Control. Release.

[34] Lasoń E., Sikora E., Ogonowski J. (1970). Influence of process parameters on properties of Nanostructured Lipid Carriers (NLC) formulation. Acta Biochim. Pol..

[35] Subramaniam B., Siddik Z.H., Nagoor N.H. (2020). Optimization of nanostructured lipid carriers: Understanding the types, designs, and parameters in the process of formulations. J. Nanoparticle Res..

[36] Fatouh A.M., Elshafeey A.H., Abdelbary A. (2017). Intranasal agomelatine solid lipid nanoparticles to enhance brain delivery: Formulation, optimization and in vivo pharmacokinetics. Drug Des. Dev. Ther..

[37] Elmowafy M., Ibrahim H.M., Ahmed M.A., Shalaby K., Salama A., Hefesha H. (2017). Atorvastatin-loaded nanostructured lipid carriers (NLCs): Strategy to overcome oral delivery drawbacks. Drug Deliv..

[38] Danaei M., Dehghankhold M., Ataei S., Hasanzadeh Davarani F., Javanmard R., Dokhani A., Khorasani S., Mozafari M.R. (2018). Impact of Particle Size and Polydispersity Index on the Clinical Applications of Lipidic Nanocarrier Systems. Pharmaceutics.

[39] Han F., Li S., Yin R., Liu H., Xu L. (2008). Effect of surfactants on the formation and characterization of a new type of colloidal drug delivery system: Nanostructured lipid carriers. Colloids Surfaces A: Physicochem. Eng. Asp..

[40] Kovačević A.B., Müller R.H., Savić S.D., Vuleta G.M., Keck C.M. (2014). Solid lipid nanoparticles (SLN) stabilized with polyhydroxy surfactants: Preparation, characterization and physical stability investigation. Colloids Surf. A Physicochem. Eng. Asp..

[41] Bhadra A., Karmakar G., Nahak P., Chettri P., Roy B., Guha P., Mandal A., Nath R., Panda A. (2017). Impact of detergents on the physiochemical behavior of itraconazole loaded nanostructured lipid carriers. Colloids Surf. A Physicochem. Eng. Asp..

[42] Penteado L., Lopes V.F., Karam T.K., Nakamura C.V., Khalil N.M., Mainardes R.M. (2021). Chitosan-coated poly(є-caprolactone) nanocapsules for mucoadhesive applications of perillyl alcohol. Soft Mater..

[43] Nnamani P.O., Hansen S., Windbergs M., Lehr C.-M. (2014). Development of artemether-loaded nanostructured lipid carrier (NLC) formulation for topical application. Int. J. Pharm..

[44] Wissing S., Müller R. (2002). Solid lipid nanoparticles as carrier for sunscreens: In vitro release and in vivo skin penetration. J. Control. Release.

[45] Notario-Pérez F., Cazorla-Luna R., Martín-Illana A., Ruiz-Caro R., Peña J., Veiga M.-D. (2019). Tenofovir Hot-Melt Granulation using Gelucire^®^ to Develop Sustained-Release Vaginal Systems for Weekly Protection against Sexual Transmission of HIV. Pharmaceutics.

[46] Wang X., Luo Z., Xiao Z. (2014). Preparation, characterization, and thermal stability of β-cyclodextrin/soybean lecithin inclusion complex. Carbohydr. Polym..

[47] Hu F.Q., Jiang S.P., Du Y.Z., Yuan H., Ye Y.Q., Zeng S. (2018). Nanostructured Lipid Carriers: A Potential Oral Drug Delivery System for Improving Drug Bioavailability. Pharmaceutics.

[48] Zielińska A., Ferreira N.R., Feliczak-Guzik A., Nowak I., Souto E.B. (2020). Loading, release profile and accelerated stability assessment of monoterpenes-loaded solid lipid nanoparticles (SLN). Pharm. Dev. Technol..

[49] Son G.-H., Lee B.-J., Cho C.-W. (2017). Mechanisms of drug release from advanced drug formulations such as polymeric-based drug-delivery systems and lipid nanoparticles. J. Pharm. Investig..

[50] Ripple G.H., Gould M.N., Arzoomanian R.Z., Alberti D., Feierabend C., Simon K., Binger K., Tutsch K.D., Pomplun M., Wahamaki A. (2000). Phase I clinical and pharmacokinetic study of perillyl alcohol administered four times a day. Clin. Cancer Res..

[51] Khan N., Shah F.A., Rana I., Ansari M.M., Din F.U., Rizvi S.Z.H., Aman W., Lee G.-Y., Lee E.-S., Kim J.-K. (2020). Nanostructured lipid carriers-mediated brain delivery of carbamazepine for improved in vivo anticonvulsant and anxiolytic activity. Int. J. Pharm..

[52] Saeedi M., Eslamifar M., Khezri K., Dizaj S.M. (2019). Applications of nanotechnology in drug delivery to the central nervous system. Biomed. Pharmacother..

[53] Zhang Q., Jiang X. (2020). Recent advances in nanotechnology-based drug delivery systems for enhanced drug penetration into the central nervous system. Nano Today.

[54] Chaves L.L., Cevolani D., Cavalcante R.S. (2021). Role of Polysorbate 80 on brain drug delivery: Evidence and advances. Eur. J. Pharm. Sci..

[55] Zhuang C.Y., Li N., Wang M., Zhang X.N., Pan W.S., Peng J.J., Pan Y.-S., Tang X. (2010). Preparation and characterization of vinpocetine loaded nanostructured lipid carriers (NLC) for improved oral bioavailability. Int. J. Pharm..

